# Perceived sustainability of the school-based social and behavior change communication (SBCC) approach on malaria prevention in rural Ethiopia: stakeholders’ perspectives

**DOI:** 10.1186/s12889-021-11216-7

**Published:** 2021-06-18

**Authors:** Fira Abamecha, Gachena Midaksa, Morankar Sudhakar, Lakew Abebe, Yohannes Kebede, Guda Alemayehu, Zewdie Birhanu

**Affiliations:** 1 Department of Health, Behavior and Society, Faculty of Public Health, Institute of Health, Jimma University, P.O. Box: 378, Jimma, Ethiopia; 2 Department of Public Health, College of Medicine and Health Science, Mizan-Tepi University, Mizan-Aman, Ethiopia; 3 USAID, Addis Ababa, Ethiopia

**Keywords:** SBCC, Schools, Stakeholders, Sustainability, Evaluation, Ethiopia

## Abstract

**Background:**

Evidence on what makes the school-engaged social and behavior change communication (SBCC) interventions on malaria prevention more sustainable are limited in literature partly due to its recent emergence. Enrolling the key stakeholders, this study explored the perceived sustainability of the SBCC interventions on malaria prevention through primary school communities in rural Ethiopia.

**Methods:**

The SBCC interventions were implemented from 2017 to 2019 in 75 primary schools and villages in rural Jimma to promote malaria preventive practices. As a part of program evaluation, this study employed a mixed-method to collect qualitative and quantitative data from 205 stakeholders following the end of the program. Data were collected using interview guides and structured questionnaires. The SPSS version 26 and Atlas ti7.1 software were used to analyze the data. Multivariable linear regression modeling was used to identify predictors of the perceived sustainability of the program (SOP).

**Results:**

The mean score of SOP was 25.93 (SD = 4.32; range 6–30). Multivariable linear regression modeling showed that the perceived risk to malaria (β = 0.150; *P* = 0.029), self-efficacy (β = 0.192; *P* = 0.003), and perceived fidelity of implementation (β = 0.292; *P* = 0.000) and degree of adoption (β = 0.286; *P* = 0.000) were positively predicted the perceived SOP. The qualitative result identified various barriers and opportunities to sustaining the program that summarized under three themes which include perceptions about the quality of program delivery (e.g inadequate involvement of stakeholders and staffs, concerns over short project life, immature sustainability efforts), school settings (e.g schools’ malaria priority, schools’ climate and quality of coaching) and the outer settings (e.g existing structures in the health and education systems).

**Conclusion:**

The study identified key predictive variables such as stakeholders’ perceived risk to malaria, self-efficacy, perceived fidelity of implementation and degree of adoption that could help to improve the sustainment of the school-based SBCC approach on malaria prevention and control. Further longitudinal study should be conducted to examine the rate of decline in program components over time and how improved sustainability would contribute to the effectiveness on malaria preventive behaviors among students.

## Introduction

Malaria is one of the top vector-borne diseases that is transmitted by the bite of infected Anopheles mosquito [1]. Globally, about 229 million malaria cases were reported in 2019, and there no significant reduction in the preceding four consecutive years [2]. For instance, the disease affected 409,000 lives in 2019 alone compared to 411,000 lives in 2018 and more than 90% of the infection occurs in Africa [2]. Nearly half (52%) of Ethiopia’s population is at risk of malaria [3]. Malaria has profound health and social effects on school-aged children affecting the critical period of learning and development [4, 5].

Malaria elimination efforts have received top priority of the global agenda by 2030 and beyond [4–7]. Essential actions such as vector control, (insecticide-treated net (ITN) and insecticide residual spray (IRS)), case management (screening and treatment), and improving the supply chain of preventive services [8] were common strategies utilized to accelerate the elimination efforts. However, applying costly strategies to eliminate malaria seems paradoxical in the context of inadequate public participation [9]. Thus, this displays the significance of intensifying sustainable multisectoral and community participatory interventions to ensure the elimination targets [4].

The SBCC approach is one of the emerging effective behavior change strategies used to advance community engagement in malaria preventive programs [4, 7]. Studies showed that the SBCC strategy effectively improved malaria preventive behaviors in the community [10, 11] and in schools [12, 13]. Ethiopia is one of the African countries striving towards elimination targets through enhanced empowerment of the community and local institutions such as schools [3, 14, 15]. Thus, the SBCC strategy was emphasized in various health strategies of Ethiopia including malaria prevention, control and elimination strategy [3, 15]. Thus, maximizing the sustained benefits of such health-promoting programs is an essential task through various participatory mechanisms [16, 17]. However, connected to its recent emergence, evidence on long-standing experiences with implementing and sustaining of the SBCC approach is limited in Africa including Ethiopia [18, 19]. Specifically, substantiation on what makes the school-based SBCC interventions sustainable is limited in the scientific literature [20].

Sustainability is defined as the capacity to maintain program services at a level that will provide ongoing prevention or treatment of health problems after the termination of major financial, managerial, and technical assistance from donors [21]. In a process evaluation sense, sustainability is considered one of the eight measures of the implementation outcomes which acts as an intermediate factor between the intervention and its effects [22, 23]. Research on program sustainability helps to understand the extent to which the interventions are being implemented in the community settings to produce the intended outcomes [24]. The sustainability indicator has retrospective implications that indicate the program process and implementation. In addition, the extent of stakeholders’ involvement and sense of ownership of the program combined with the quality of implementation (fidelity) greatly determine the sustainability of a program (SOP) [17].

Due to its very participatory nature, efforts to understand the sustainment of the school-based SBCC approach on malaria prevention requires exploring or examining the extent of stakeholders’ engagement, sense of ownership and commitment to sustain it in the future. The SOP is usually measured in objective terms based on the monitoring data collected from specific program components and activities meant to be sustainable. Subjective measures of stakeholders’ perceptions towards the SOP is also considered an alternative indicator of process outcomes [25]. However, empirical evidence on stakeholders’ perspectives regarding SOP is limited in scientific literature as far as the SBCC approach is concerned [23].

Therefore, enrolling key stakeholders, this study explored the experiences and perceived sustainability of the SBCC approach on malaria prevention through primary school communities in Jimma, rural Ethiopia. A mixed-method design was applied in this study to enable collecting and analyzing data from multiple sources to understand the issue under the study in a broader context. The quantitative design was aimed to measure the perceptions of the stakeholders about the implementation and sustainability of the program, while the qualitative one was used to explore the associated experiences and perspectives.

## Methods and materials

### Study area and contexts

The school-based SBCC intervention on malaria prevention and control was implemented in 75 rural primary schools and respective villages selected from five districts of Jimma, Ethiopia, from 2017 to 2019. Jimma is one the zones found in Oromia region located 352 km away from Addis Ababa, the capital city of Ethiopia. Based on the projected 2007 Census conducted by the CSA, the total population of Jimma was 2,486,155, of whom 50.3% is male and 49.7% are female. While 137,668 or 11.31% are urban inhabitants, the majority of the population was rural residents. The zone lies with an altitude ranging between 900 to 3500 m above sea level.

Having characterized by stable malaria transmission rate, about 85% of population of Jimma (i.e. 73% of the villages in Jimma) is at risk malaria. According to the health department report of Jimma, a total of 11,259 malaria cases were reported in Jimma in 2017 (a year before the program launch). Engaging primary schools, the current intervention was conducted in five selected districts of Jimma characterized by high malaria transmission rates. An annual parasite incidence (API) rate in 2017 was 16% in Chora Botor, 14.1% in Shebe Sombo, 10% in Nono Benja, 5.5% in Limmu Kossa, and 3.1% in Gera; according to the Zone’s health department. The five districts are Limmu Kossa (population = 209,261), Shebe Sombo (population = 146,805), Nono Benja (population = 77,452), Chora Botor (population = 74,756), and Gera (population = 147,120). Data was collected during the period between May 10 to 30/2020.

### Description of the school-based SBCC intervention

Guided by the SBCC theoretical principles, various educational, communication, and capacity building activities were implemented for a period of 2 years (i.e. from 2017 to 2019) to empower schools, stakeholders and the local community on malaria preventive actions. It was implemented in 75 schools and respective villages in selected malaria-endemic settings. More specifically, the interventions were designed to promote the five key malaria prevention and control practices both in schools and at community levels. These were the use of insecticide nets (ITNs), appropriate & timely seeking care for malaria, appropriate use of quality anti-malaria drugs, acceptance of insecticide residual spray (IRS), and mosquito breeding habitat modifications such as gardening and solid waste management as part of vector control efforts.

The program was initiated through the consultations of various stakeholders from the target community that include key professionals from health departments, education offices, health extension workers (HEWs), and school teachers. The supervisory committee was established to conduct sound and participatory malaria situation analysis, the community needs assessment, and devising intervention strategies. Accordingly, heads of health departments, education offices, school principals, malaria focal persons (school teachers), and health extension workers (HEWs) received first-level training on community mobilization and malaria prevention and control programs. Under the supervision of health departments, trained school principals and teachers were encouraged to cascade down the training to peer educators through one-to-five peer learning networks among school students. One-to-five peer learning networks is a social network that consists of 6 individuals (1 leader and the other 5 members) in one group. The purpose is to promote supports among students through a peer learning network.

Peer education activities at school were facilitated by trained peer educators under the supervision of the teachers and school principals. All schools students were reached by malaria education through peer education before they were sent to teach their parents, neighbors, and community members. The peer education activities were aided by guides and various health learning materials (HLMs) such as flip charts and leaflets. The process by which students were first trained and educated on malaria and peer education strategies then after guided to transfer the information to parents and the local community was termed in this intervention as the peer learning and education approach (PLEA). Various educational and communication activities such as social dramas, campaigns, and role-plays were conducted within schools and in the nearby communities.

Rigorous supportive supervision, field visits, and review meetings were implemented in participatory ways to build sustainability and ownership of the SBCC activities. The intervention was integrated into the existing primary health care activities and therefore, the HEWs were actively collaborated in the SBCC activities to cascade it to the household level through women-centered health development teams (HDT) and larger community groups to ensure long-lasting sustainability. The HEWs are paid community health workers who have roles of public health education and behavior change communications on sanitation and preventions of common infectious diseases such as malaria, TB, HIV/AIDS, and maternal health services [14]. However, the HDTs are unpaid women volunteers who have the capability to carry out community mobilization tasks such as immunization campaigns, keeping track of pregnancies and illnesses for up to 30 households in the neighborhood, and in collaboration with the HEWs [26]. Details about the school-based SBCC interventions were presented in a study published somewhere else [12, 13].

### Study design

As part of the school-based SBCC program evaluation, the current study employed a mixed research design to collect post-intervention data following completion of the program. The mixed-method design was applied to enable collecting and analyzing data from multiple sources to broadly understand the issue under study. The quantitative design was aimed to measure the perceptions of the key stakeholders about the implementation and sustainability of the program, while the qualitative one was used to explore the associated experiences and perspectives. The experiences and perspectives of the participants on the program implementation process, sustainment, and other broader contexts were explored based on the major domains or propositions identified in quantitative research objectives [27]. The two research approaches were supposed to complement each other in which one method would compensate for the limitations of the other one. Finally, findings of qualitative research part were used to interpret or explain the findings of the quantitative study for a better understanding of the problem.

### Quantitative method

#### Study population and sample size

Participants encompass key stakeholders including officials from education and health offices, community health extension workers (HEWs), school directors, school malaria focal teachers and the program’s field officers. Stakeholders are defined in this study as, those individuals who received basic and ongoing training on the program contents, program delivery, and monitoring process of the SBCC interventions on malaria prevention and control. They were empowered, supported, and have given full responsibility to plan, implement and monitor the malaria prevention communications in their respective schools and villages.

There were about a total of 209 key stakeholders who have received training on the management, monitoring, and dissemination of the school-based SBCC intervention and who have been facilitating and supervising the program in collaboration. This includes 170 participants from schools directors and schools malaria focal teachers in 75 schools, 10 health professionals from health offices in five the districts, and 39 community HEWs. A total of 205 (out of 209) key stakeholders were included in the survey that was conducted following the completion the intervention.

#### Data collection instrument and techniques

Data were collected using a structured questionnaire adapted from relevant literature on program implementation outcomes and the sustainability [23, 28–31]. The questionnaire covered items addressing the socio-demographic characteristics, knowledge on essential malaria actions (EMAs), threat perceptions (risk and severity) regarding malaria, self-efficacy or competence, perceptions of community supports and schools climates. Further, the psychometrically measured key implementation outcomes such as fidelity of implementation (FOI), degree of program adoption, sustainability of a program (SOP) were covered. The questionnaire was developed in English and translated into *Afan Oromo*, the local language to facilitate understanding. The stakeholders were contacted at their workplaces and they encouraged filling the questionnaire.

#### Measurements and scoring

##### Knowledge of essential malaria actions (EMAs)

The EMAs elicited knowledge regarding the eight key malaria actions addressed in the project. The respondents were asked to mention the eight EMAs, on an open-ended format. All right responses were counted to create composite scores that ranged from *“0 = zero knowledge” to “8 = highest knowledge”.*

##### Perceived risk to malaria

Perceived risk to malaria is defined as perceptions about the likelihood of acquiring malaria. Five items were used to measure this construct on a five-point Likert scale which ranges from *completely disagree = 1* to *completely agree = 5.* This scale produced reliable measures with an acceptable level of internal consistency (α = 0.70). Reverse scoring was done for negatively worded statements before analysis.

##### Perceived malaria severity

The perceived severity of malaria is defined as perception about the bad consequences of malaria infection such as severe illness, death, or interruption with their daily works and performances. This construct was measured by four items (α = 0.61).

##### Self-efficacy/competence

This was defined as belief about the psychological empowerment (i.e. perceived competence) related to a specific issue (i.e. about malaria and the school-based SBCC approach in this case) [32–34]. Eight items were used to measure the perceived personal competence, (α = 0.90). Composite scores were used in the analysis.

##### Perception about schools climate

This was conceptualized as stakeholders’ perceptions about the extent to which the organizational system and social supports within the school settings are responsive to the SBCC program and their expectations. These include perceptions towards school systems, social relations, decision making process and support that influence the program implementation. The measure was adapted from the *organizational climate scales* and designed specifically to measure the stakeholders’ perceptions in the context of the SBCC approach on malaria prevention [35]. *The exploratory principal component analysis (EPCA) yield eight valid items (*α = 0.83) and the composite score was used in the subsequent analysis.

##### Perceived community support

This taps the perceptions about the existing community such as sense of belongings (connections), community participation (active participation in community activities) and community organizations (perceived social support of in the community) that can provide opportunities for members of the community to participate in malaria preventive actions. Nine (9) items (α = 0.77) with responses rated on a 5-point scale from (1) strongly disagree to (5) strongly agree were used to measure the perceived community supports [36]. High composite scores were interpreted as a higher importance of this factor.

##### Perceived fidelity of implementation (FOI)

Fidelity of implementation (FOI) is defined as individuals perceptions about the degree to which an intervention is delivered as intended [31]. FOI has direct relation with outcome of an intervention as a mediating variable between context and intervention effectiveness [37]. The perceived FOI was measured by the stakeholders’ self-rating questions on five-point Likert scale. Nine items (α = 0.89) were adapted from previous study [37] and designed based on the major SBCC program components and activities.

##### Adoptions/degree of readiness to adopt

Adoption is defined as the degree of uptake or actual use of a program [23]. For this study, scale consisting of five items (α = 0.72) were adapted from previous studies [23, 38] to measure the degree of adoption or readiness to adopt the school-based SBCC interventions on malaria preventions. Items were designed using the five point Likert scale format. Composite was constructed with higher scores indicating high degree of adoptions or uptake by the stakeholders.

##### Perceived SOP

The perceived SOP was defined in this study as, the stakeholders’ perception about how likely the school-based SBCC interventions on malaria preventions will be continued functional in the area. It was measured immediately after the completion of the program from the stakeholders’ perspectives using items that were adapted from the *sustainment measurement system scale* [39]. Dimensions such as perceived community supports, organizational capacity and system supports of hosting organization (i.e. schools in this case) and stakeholders capability to run the program were assessed [40]. A total of 6 items ((α = 69) designed on five-point Likert scale were used in which the stakeholders are asked to rate their opinion across range of factors that affect the sustainability of this program.

### Statistical data analysis

Quantitative data were analyzed using SPSS 26.0 software. Descriptive statistical measures such as frequency, mean and proportion were calculated. The Pearson’s correlation analysis was carried out to examine the relationship between the perceived SOP and other psychometrically measured variables. Similarly, an independent sample t-test was carried out to explore associations between the perceived SOP and other dichotomous variables.

Multivariable linear regression modeling was performed to identify the independent factors associated with the perceived SOP of the school-based SBCC approach on malaria prevention. Standardized regression coefficients (β) were interpreted to understand the independent effects of the selected predictors on the outcome variable. *P*-value less than 5% were considered for significant association.

### Qualitative research part

#### Qualitative method participants

Participants for qualitative method were selected based on prior knowledge on the program experiences, exposure to the interventions and contexts [27]. Thus, purposive sampling technique was employed to recruit informants who were actively engaged in the program as lead stakeholders. Various segments of stakeholders were reached out with the ultimate goal of maintaining maximum variability assumptions [41] The intent was to address multiple perspectives, experiences, and factors influencing the program sustainability. Accordingly, nine key informants or key stakeholders who had administration roles in the project were selected. Furthermore, document review was conducted to collect secondary data on activities and review meetings reports regarding the school-based SBCC interventions.

#### Qualitative method tools and data collection

The qualitative data was collected using guides that were developed based on the contents and propositions of the quantitative studies. The guide has list of few interview points with several follow up probes used to capture beneath of the issue. The interviews were conducted in quiet spaces found in the back yard of each school and some interviews were conducted in quite offices in the schools. The interview process was interactive and participatory facilitated by experienced interviewers. Data collection was assisted by audiotape records and the data collectors took field notes. The average recorded time to run the interview was 45 min.

#### Qualitative method data analysis

The recordings and field notes were transcribed verbatim and translated into English. The Atlas. ti 7.1 software for analysis was used to guide the analysis process. Data analysis was done through the thematic analysis process to build conceptually defined themes [41]. Initial analysis was started with quick identification and assigning of codes to descriptive data segments based on pre-defined research questions or prepositions identified in research objectives. Further coding was done to clear out any relevant emerging segments that require assigning new codes and alternative explanations. Coding was done independently by two experienced public health researchers. Besides, quotes of participants’ expressions that exemplify key concepts were used directly during analysis and interpretation. Finally, the results and discussion are presented by triangulating the qualitative and quantitative findings.

## Results

### Socio-demographic characteristics

Two hundred and five stakeholders were participated in the study making the response rate of 98.10%. The majority of the participants were teachers in a profession that accounts 137 (66.83%). Male participants were 130 (63.41%) and 109 (53.17%) participants had age ranging between 25 and 29 years old. Large number of participants have Oromo ethnic background with 190 (92.68) and protestant and Muslims account major religions with 78 (38.05%) each. The majority of the participants were married, 173 (84.39%) and 95 (46.34%) have monthly salary of 4000-4999birr. Participants who reported diploma as the highest level of education attained and experience of 6–10 years account majority with 150 (73.17%) and 126 (61.46%) respectively. A large segment of the participants 119 (58.05%) was also reported having received some sort of health-related training other than that of the current project (Table 1).

**Table 1:** The relationship between the key stakeholders’ socio-demographic characteristics and perceived sustainability of program (SOP), Jimma, 2019, (*N* = 205)

	Frequency (%)	SOP (M ± SD)	*F-statistics (df)*	*P*-value
Districts				
Gera	40 (19.80)	24.68 ± 4.59	3.52 (df = 4)	0.008
Shebe Sombo	41 (20.29)	26.95 ± 4.20		
Nono Benja	38 (18.81)	24.51 ± 4.65		
Botor Tollay	40 (19.80)	26.95 ± 3.92		
Limmu Kossa	43 (21.29)	26.68 ± 3.74		
Job category				
Teachers	137 (66.83)	25.99 ± 4.47	−0.29 (df = 203)	0.384
Health workers	68 (33.17)	25.81 ± 4.04		
Age				
< 24 years	11 (5.37)	27.27 ± 3.58	0.69 (df = 3)	0.560
25–29 years	109 (53.17)	25.99 ± 4.59		
30–34 years	72 (35.12)	25.51 ± 4.05		
> 35 years	13 (6.34)	26.62 ± 4.17		
Gender			*t (df)*	
Male	130 (63.41)	26.07 ± 3.86	0.34 (df = 203)	0.735
Female	75 (36.59)	25.85 ± 4.58		
Ethnicity				
Oromo	190 (92.68)	25.83 ± 4.41	−1.24 (df = 203)	0.110
Others^a^	15 (7.32)	27.27 ± 2.87		
Religion				
Orthodox	49 (23.90)	26.20 ± 4.32	0.63 (df = 2)	0.536
Muslim	78 (38.05)	26.19 ± 3.82		
Protestant	78 (38.05)	25.50 ± 4.80		
Marital status				
Single	32 (15.61)	25.70 ± 4.36	−1.75 (df = 203)	0.569
Married	173 (84.39)	27.16 ± 3.94		
Level of education				
BSc degree	55 (26.83)	25.71 ± 4.73	−0.45 (df = 203)	0.277
Diploma	150 (73.17)	26.01 ± 4.18		
Experiences				
< 5 years	21 (10.23)	26.24 ± 3.66	1.97 (df = 2)	0.142
6–10 years	126 (61.46)	26.32 ± 4.60		
> 11 years	58 (28.29)	24.98 ± 3.81		
Monthly salary				
< 2999birr	34 (16.59)	26.88 ± 4.13	0.88 (df = 3)	0.455
3000-3999birr	37 (18.05)	26.22 ± 4.12		
4000-4999birr	95 (46.34)	25.68 ± 4.53		
> 5000birr	39 (19.02)	25.44 ± 4.15		
Received training on a health-related issue				
Yes	119 (58.05)	26.25 ± 4.19	−1.25 (df = 203)	0.224
No	86 (41.95)	2548 ± 4.48		

Key: ^a^Others = Amhara, Kafa, Tigre, Guraghae, *SOP* sustainability of a program, *M* mean, *SD* standard deviation, *df* degree of freedom

### Descriptive statistics and Pearson’s correlation coefficients

The mean score of the perceived SOP was 25.93 (SD = 4.32; range = 6–30) which was considerably higher score. This implies the stakeholders hold a perception that the school-based SBCC approach on malaria prevention is more likely to sustain in the schools over the coming time.

Furthermore, Pearson’s correlation coefficients (*r*) showed that the majority of the psychographic constructs were significantly and positively correlated with SOP; except perceived severity and school climate scores. The highest and lowest positive significant correlation was observed between FOI and SOP (*r* = 0.45; *P* < 0.01), and between perceived risk and SOP (*r* = 0. 14; *P* < 0.05), respectively (Table 2).

**Table 2:** Descriptive statistics and Pearson’s correlation (r) for measures of the measured the perceived FOI, adoption, SOP and related constructs, Jimma, 2019 (*N* = 205)

SN		1	2	3	4	5	6	7	8
1	Competence/self-efficacy	1							
2	Perceived malaria severity	0.05	1						
3	Perceived malaria risk	−0.01	0.42^b^	1					
4	Perceived community support	0.01	−0.01	0.03	1				
5	Perceived school climate	−0.10	−0.05	0.18^a^	0.08	1			
6	Perceived FOI	0.32^b^	0.24^b^	0.10	0.20^b^	−0.19^b^	1		
7	Perceived degree of adoption	0.34^b^	−0.07	−0.07	0.15^a^	0.09	0.33^b^	1	
8	Perceived SOP	0.36^b^	0.06	0.14^a^	0.15^a^	−0.04	0.45^b^	0.43^b^	1
9	Number of items	8	5	4	4	8	9	5	6
10	Scale range	8–40	5–25	4–20	4–20	8–40	9–45	5–25	6–30
11	Means	35.28	14.33	14.29	7.12	31.22	33.50	17.76	25.93
12	Standard deviations	4.37	3.05	4.34	1.95	6.73	6.92	2.55	4.32

Key: ^a^ Correlation is significant at the 0.05 level (2-tailed). ^b^ Correlation is significant at the 0.01 level (2-tailed).

#### Predictors of the perceived SOP

The multivariable regression modeling demonstrated a significant relationship between the selected variables (during the bivariate analysis) and the perceived SOP score. Accordingly, personal factors such as the perceived risk of malaria (standardized coefficient; β =0.150; *P* = 0.029), self-efficacy towards program implementation (standardized coefficient; β =0.192; *P* = 0.003) were demonstrated positive effects on the perceived SOP. This implies; those stakeholders who have higher perceptions of malaria risks and perceptions about the ability to implement the school-based SBCC program think that the program will sustain in the future.

On the other hand, the analysis identified that the program-related dimensions such as perceived FOI (standardized coefficient; β =0.292, *P* = 0.000) and perceived degree of program adoption (standardized coefficient; β =0.286; *P* = 0.000) positively predicted the perceived SOP among the stakeholders. The idea is that, stakeholders who believe that the program was implemented very well think that higher probability to sustain it. Similarly, those who reported a higher degree of adoption hold a strong belief that the program would be implemented effectively in the future. However, none of the community settings and school climate measures were predicted the perceived SOP in this study. The highest and lowest potential predictors of perceived SOP were the perceived FOI (effects = 29.2%) and perceived risk to malaria (effects = 15%) (Table 3).

**Table 3:** Unstandardized and standardized coeffs for the direct predictors of the perceived SOP on the school-based SBCC approach on malaria prevention in Jimma, 2019, (*N* = 205)

Predictors	Unstandardized coeffs., (β)	Standardized coeffs., (β)	*P*-value
Gender (male)	0.107	0.012	0.862
Age in years	0.084	0.066	0.325
Educational level (diploma)	−0.466	− 0.048	0.467
Work experience in years	− 0.117	− 0.076	0.209
Knowledge on EMAs	−0.004	− 0.033	0.585
Self-efficacy towards the program	0.190	0.192	0.003
Perceived severity of malaria	−0.064	− 0.045	0.509
Perceived risk to malaria	0.149	0.150	0.029
Perceived community support	0.088	0.040	0.537
Perceived school climate	−0.011	−0.012	0.853
Fidelity of implementation (FOI)	0.182	0.292	0.000
Degree of adoption of the program	0.485	0.286	0.000

#### Results of the qualitative study

The qualitative result identified three major themes and respective sub-themes regarding the perceived barriers and opportunities to adoption and sustaining of the school-based SBCC intervention on malaria prevention. Theme 1: perceptions about program coordination and delivery that include inadequate involvement of key stakeholders and relevant health staffs, unprecedented turning over of the trained staffs, concern over short project life with immature sustainability efforts, flawed program monitoring and supervision, and low community promotion of the program to improve sense of ownership. Theme 2: perceptions about the inner organizational/school settings such as low schools priority, supportive schools climate, and coaching of the program. Theme 3: perceptions about the outer settings or community supports such as the existing health system, education sector structures, and community social networks.

Accordingly, the majority of the key informants mentioned that the lack of engaging key stakeholders such as village councils, political leaders who have the power to influence and mobilize the community, and the health centers (who are expected to have the authority to supervise the HEWs through the structure called primary health care unit (PHCU). Confusions in the roles and responsibilities about “who should do what” and “who should owe the program” among the stakeholders (between the schools and health sectors) were perceived as challenges to sustainability. Lack of engaging all possible and relevant staffs combined with the unprecedented turning over of these trained staffs, the short project life, immature sustainability efforts, and interrupted technical and material supports were pressing concerns of the stakeholders that might affect the continuity of the program. They mentioned that low community promotion of the program has affected the sense of ownership which might in-turn determine its sustainability.

The result also identified that the low school’s priority of a program, teachers’ skeptic behaviors on coaching of the school-based SBCC program as factors related to the inner organizational/school settings. However, they acknowledged that the benefits of the existing structure in the education system in facilitating the implementation and institutionalization of the SBCC program into the school system. At the school level, the structure was locally called *“geengoo barattootaa”,* which mean; “the student circle”. The circle consists of five members and a leader. It was aimed at improving students’ academic performance in primary schools by facilitating peer learning activities.

Finally, factors related to the outer settings (structures in the health and education system, community networks) were identified as opportunities to sustain the program. The existence of a formal structure (school networks) in the education system called “cluster resources center” [CRC] and the PHCU of the health sector combined with their commitment to work in collaboration on malaria prevention has provided a great opportunity for sustainability. The CRC is an inter-school network aimed at enhancing educational quality within the cluster (Table 4).

**Table 4:** Summary of themes, sub-themes and selected quotations on barriers/challenges and opportunities of the school-based SBCC implementation and adoption in Jimma, Ethiopia, 2017–2019

Themes/subthemes	***Selected quotations***
**Perceptions about the program coordination and delivery**	
Lack of involvement of key stakeholders in the program	*Mobilizing the community is possible and effective if and only the village councils are involved in malaria training and be able to feel malaria as a health priority.* ***A HEW*** *Though health personnel working in the health centers have great roles in ensuring community health; no health centers were involved in the current program. None of them were invited to that program. But the HEWs are basically supervised by health center directors; as part of the primary health care unit structure. So it would be good to engage representatives of health centers, at least a director and HEWs supervisor.* ***A health officer*** *We were in the program since its inception. We were consulted for how to investigate the local malaria situation. We got the training about malaria and how to implement the program. We received material supports such guides.* ***A school director***
Inadequate staffs, limited training and unprecedented turning over of the key staffs	*What is concerning is problem … is the sustainability issue. Sustainability is always questionable. The most important reason is the unprecedented turning over of trained teachers who were nominated as focal person of malaria education from schools. Following the approval staffs transfer policy that occurs once every year, there were unprecedented transfer of teachers from one school to the other schools or places. As a result, the schools are subjected to losing the trained and experienced personnel who could play big roles and take responsibility to support sustainability of the program in the schools.* ***Health officer*** *Nowadays, the program has effectively been implemented only in few schools. There is no strong control in some schools where the key program coordinators have been transferred. The reason for this is turnover of trained teachers and HEWs. For instance I myself may stop working on this program if I am transferred to other villages with relatively low malaria incidence and similarly; another HEW who assigned here, in place me; cannot continue doing the program because she was not trained on the issue.* ***A HEW*** *It was only one school that has got a chance to participate in this program among two schools in our village. Now; some teachers from the participating school were transferred to other place. Had the two schools in our village were participated in the program, trained teachers in the remaining school would effectively continue implementing the program. Even, I am not sure whether the program will be kept implemented if I left for further education.* ***A teacher, school malaria focal person***
Perceived adequacy of project life and immature sustainability process	*Initially, at the start of the program, it was planned that the program will be implemented for more than 2 years and above. But, they [project owners] abruptly discontinued the program before it was institutionalized around its ending while we were implementing it. These things are some of the challenges that were impeding the continued implementation and this may ultimately affect the intended change.* ***A health officer*** *I mean, some problems of that project … from the very beginning, it seems not a big project but something like pilot study. It was planned for many years but they didn’t stay more than 2 years. There was nothing done to ensure sustainability. No direction was set; as far as am concerned. As the program life nears its end, they [project owners] would have to discuss with political bodies, districts leaders and representative of the community to put direction the way this project can be sustained and to give responsibility to the education and health offices to keep performing.* ***School Director*** *Though we had good lesson from the program, We cannot say it was exactly implemented as it was planned. Even though it was not long lasting as the intended project’s life, its implementation was found somehow ongoing. At the beginning time of project’s life, there were efforts such as community teaching; monitoring and coordinating communication activities. But now; except at schools’ club level, it is not being implemented the same way. Nevertheless, we cannot claim that the malaria prevention program is completely forgotten in the school.* ***A health officer*** *The trained senior students can also leave the schools for further higher education where there is no such program and support. The newly coming students have no training on such program and this could create gaps to continuing the school-based malaria prevention education. But, if the whole schools and health professionals in the districts were involved, there won’t be interruption as the newly coming students and staffs can keep implementing the program because they were familiar with it.* ***A HEW*** *As I have mentioned earlier, connected with accessibility … previously there was continuous supply of stationery materials like large sized paper for printing and displaying of the key malaria messages, parkers used during peer education sessions and sport clothes with designed malaria messages or slogans which has been used during campaigns. Now, these are all interrupted. Though the school-based malaria program exists today in our school, it’s being considered top agenda for everyone unlike the previous time.* ***School Director***
Perceptions about program coordination, monitoring and supervision	*In fact …, some of the challenges are attributed to the poorly managed inter-sectoral collaboration. For instance, the schools are regularly reporting the PLEA-malaria activities to the district’s education offices where they document the data (without further reporting the activities to health offices and the health office without asking for it as well). Though the presence of this club (the PLEA club) ideologically linked with the concept of the inter-sectoral collaboration of health, education, and agriculture; it’s noted that the commitment to take initiative to coordinate the activities to provide regular feedbacks was low.* ***Sustainability review meeting report*** *The tasks health extension workers perform …, the tasks education offices perform …, the tasks health office performs …; were all not well organized at that time. Everything has been implemented in different ways.* ***A health officer*** *Some [district’s] health office personnel claim to get no reports [from school-based project coordinator] about what is going in the schools pertaining to malaria. In fact, the health office also did not taking initiative to gather reports and use the data for routine planning, monitoring, and evaluation of malaria communication efforts.* ***Sustainability review meeting report*** *The health offices neither have a checklist of malaria activities to execute at school the ACP project school-based malaria activities. In fact, they even lack follow-up checklists of the community-based malaria education and preventive activities.* ***Sustainability review meeting report*** *In our experiences, we have learned that it is a trend that we often warmly implement an intervention under strong control at the first time. But, we gradually get fed up, loss commitment and in some worst cases, stop it. However, if there is a continued supervision and request for reporting of the designated activities, there is no question that the implementation will be continued. The more closely the supervision by the concerned bodies, the more likely the interventions is continuously implemented. A* ***HEW***
Inadequate promotion of the program to the community for improved sense of ownership	*It’s [the program] was not well promoted to the level of communities. Look, giving training for only schools might affect the community to improve attitude or get the support of the community. Many more people must be trained.* ***A school director***
**Inner organizational settings (schools and health sectors)**	
Perceptions about schools priority, climate and coaching of school malaria program	*Some teachers and our co-workers [HEWs] often say, ‘… I am assigned here to teach and not to care or treat [to deal with health issues]. In fact, I am not trained on how to deliver such a program. So, I don’t think I am responsible …* ***A HEW*** *I think the health extension workers can do it. Since it [malaria prevention education] is a routine task of the HEWs, they have to take initiative and organize campaigns, and may request school support later on. They are in the community to teach about malaria.* ***A teacher*** *“Some teachers say, ‘we are assigned here for teaching, I am working in different structure in the school, I am assisting students learning, I have my own tasks that I am responsible to perform and other’* ***A school director*** *There is some confusion about who should be the owner of the program. Some schools think it belongs to health sectors and they wait till the health workers come to them to initiate and do it.* ***A school director*** *Teachers acquired not only knowledge about malaria, but also skills needed to guide and implement the PLEA-malaria to sustain the practices* ***A school director***
**Perceptions about the outer settings (health and education system structures)**	
Existing structures in the health and education system, community networks as opportunity to sustain	*Malaria prevention and control is one of the top health extension packages and so activities of the HEWs. And, so it will be easy for them to implement the school-based SBCC intervention on malaria. The HEWs are accountable to primary health care unit (PHCU) supervisors.* ***A health offices director*** *During the campaign events such as immunization days, we contact and inform it to the health development team [HDT]. Then, they will mobilize households in their catchment area. We can also implement malaria prevention activities or campaigns on issues such as IRS promotion together with the women’s army.* ***A HEW*** *Availability of various health and health related clubs such as anti-malaria, anti-HIV/AIDS, environmental protection and conservation, sport club …*) *in schools, though it wasn’t closely supported and owned by the health offices. Furthermore, the existence of formal schools networks called “cluster resources center” [CRC]. With the ultimate goal of enhancing educational quality, the CRC has coordination unit at district education office level to facilitate monitoring and supervision of the teaching-learning activities in schools within the cluster. So, the health offices (PHCU), the education office (CRC), and the student level social network and their commitment to work in collaboration on malaria prevention has provided a great opportunity for sustainability of the program.* ***Sustainability review meeting report*** *We have a joint network at the school leve,; called “BARNOOTA WAL-MADDEESSA”; [which mean pupil-centered education]. It consists of students, teachers, and parents that extend in the hierarchy of the education system (from top to school level). It was established for the purpose of improving the quality of education in primary schools. And, I think it’s an opportunity to implement such a program on malaria.* ***A school director***

*Key*: *HEW* health extension workers, *ITN* insecticide treated nets, *IRS* insecticide residual spray, *SBCC* social and behavior change communication, *PHCU* primary health care unit, *HDT* health development team, *PLEA* peer learning and education approach, *ACP* advancing community practice, *CRC* cluster resources center

Similarly, a document review result showed that basic training was offered for 189/225 (84%) school teachers, school principals, and other key stakeholders. School peer educators among students. A total of 13,400/8842 (66%) peer leaders were trained as key facilitators of peer education, and these leaders reached 37,824/40,000 other students. Further, various school-level clubs such as anti-malarial and mini-media clubs were established, strengthened, and networked in all 75 (100%) schools. The project has reached 54, 412 households using trained students as change agents and messengers of malaria information. Besides, more than 120, 434 people were received essential malaria actions through various approaches. The project carried out about 475/298 (62.7%) small and large-scale community campaigns.

The project rendered about 74.3% (390/525) supportive supervision, 60% (15/25) review meetings, and 123.3% (2220/1800) visits of local households to monitor and track malaria-related preventive behaviors. Malaria-specific clubs are established and performing malaria preventive tasks in all 75 schools. Guided by the steering committee, functional linkage was created between education, health departments, and community health workers (HEWs) to ensure the sustainability of the program. Malaria preventive activities such as peer education, parent education, and campaign monitoring and reporting checklists were distributed to all schools. Furthermore, various communication materials such as leaflets/brochures; (56, 022), posters; (3297), flip charts; (1337), and training manuals/educational guides, (1969) were distributed to all targeted schools to support SBCC activities at the local level.

## Discussion

The current study examined the stakeholders’ perceptions towards the sustainability of the SBCC on malaria prevention through school communities. The study applied a mixed research method to explore the perceived SOP and identify predictive variables used to sustain the school-based SBCC intervention on malaria prevention. The result showed that perceived malaria risk, self-efficacy, perceived FOI, and perceived degree of adoption were positively predicted the perceived SOP among the key stakeholders.

The result showed that there is a positive perception that the program would be continued implemented in the schools over the coming years. Self-efficacy was identified as one of the predictive variables to help sustain the program in this study. Accordingly, perception of competence towards the school-based SBCC on malaria prevention was positively associated with perceived SOP. Literature showed that health program sustainability is affected by the characteristics of the implementing individuals in addition to the organizational and community supports [17, 24, 42]. Specifically, the knowledge and skills of employees towards the program affected their beliefs about its suitability [43, 44]. It was reported that the quality of staff training and motivation schemes provided could empower the stakeholders for future performance [30]. For instance, an evaluation study of an intervention to reduce aggressive behavior in schools found that the provision of adequate training for school coordinators and teachers facilitated sustainability [45].

In this study, the higher perception of risk to malaria infection positively predicted the perceived sustainability of the school-based SBCC intervention. Threat perception is a potential factor that drives and motivates individuals towards implementing the threat alleviating efforts and this might have brought a commitment of the stakeholders towards a continued implementation of the program. Behavior change theories indicated that people are more motivated to approve and participate in health-promoting or disease preventive behavior when they perceive that they are vulnerable to the disease or problems [46, 47]. The study area is one of the malaria-endemic settings and this might be the reason for these positive relationships. However, it was reported that malaria incidence rates are showing a considerable reduction in Ethiopians which appears to influence public perceptions as well [9, 13]. The result of this study implicates that the need to consider individuals’ disease perceptions while dealing with program sustainability issues.

The perception stakeholders’ towards the FOI positively predicted the SOP in this study implying that promoting implementation delivery would contribute to sustainability in the future. A consistent finding was reported in previous studies that programs with higher levels of fidelity and adaptability are likely to be sustainable [16, 48]. The qualitative result identified that the program delivered an acceptable level of critical components such as training, supervision, review meetings, and peer educations. For instance, 84% of the intervention’s delivery was recorded for training activities and 60% for review meetings. Literature showed a similar result in that a minimum intervention dosage or threshold of 62% can lead to the desired outcomes [49]. The existence of discrepancies between the dosage intended and dosage offered was reported in previous literature [49].

The positive relationship between the FOI and SOP observed in this study might due to an improvement in the level of acceptability of the intervention by the stakeholders and community. This indicates the connection that exists between program fidelity and sustainability implying the need to emphasize sustainability in the early phases of program processes such as design and implementation [50]. This calls for proactive thinking for careful designing of the program components such as training, partnerships, engagement, inter-personal relations in order to enhance the continuation of the program. Evidence showed that the adoption of community-based interventions depends on the extent of community and stakeholders’ involvement and acceptance which in turn influence sustainability [40]. The effect of adoption on sustainability might be mediated by the degree of the intervention’s fit to local settings, needs, and policy (i.e. extent of feasibility and acceptability). This fact was shown in previous studies that school health program that was well integrated into school policies, plans and aligned with time available for schools, matching to schools’ communities needs to be sustained after the intervention [17, 51].

Notably, a previous un-published survey stakeholders’ conducted on the same program (i.e. the school-based SBCC intervention), showed that a high level of acceptability and feasibility of the program (Abamecha et al: Perceived implementation outcomes of the social and behavior change communication (SBCC) approach on malaria prevention through schools communities in Ethiopia, unpublished). The fact that the adoption score positively predicted the SOP in this study can be explained in many ways. First, the existence of systems and structures in the education and health systems that could promote adoption and this would in-turn the sustainability of a program. A qualitative result indicated that the existence of a formal structure in the education system called CRC and the PHCU of the health sector has provided great opportunities for sustainability. The CRC is defined as an inter-school network aimed at enhancing educational quality within the cluster [52]. Secondly, considerably higher adoption rate (100%) of critical project components such as creating health-related social networks in schools such as anti-malarial, mini-media, and sports clubs were established in all 75 schools and all the targeted schools were undertaking peer and parent education on malaria with a varying schedule that ranges from every to every 3 weeks. Thirdly, this relationship might also be moderated by the amount of perceived FOI among the stakeholders. Thus, further research is required to understand how fidelity and adoption of the program interacted to influencing the SOP.

In the current study, though the measures of community supports and school climate were not significantly associated with the SOP, the majority of the qualitative participants were described that the existing school system/structure as an opportunity to sustain the program. The key informants acknowledged that the existing structure in the education system locally called “*geengoo barattootaa*”; to mean “student’s circle”, helped them to facilitate the implementation and institutionalization of the SBCC into the school system. The student’s circle consists of five members and a leader which is aimed at improving the quality of education in primary schools. However, a previous study suggested that a positive school climate was associated with the continued implementation of school-based health intervention [53]. In addition, inadequate staffing and administrative support hindered the sustainment of school-based programs [54, 55] .

The finding from the qualitative study also reported some challenges to the program such as lack of involvement of community leaders (community councils and village leaders) who have power and credibility to mobilize the community, and low school priority of the program, weak coaching, and skepticism from some teachers. Furthermore, the turning over of the trained staff was one of the serious concerns of the stakeholders that could affect the sustainability of the current study. A previous qualitative study reported that low turnover of staff is associated with pursuing more knowledge and experience with the programs than those with a higher turnover [43, 44]. This might be, new decision-makers did not always share the enthusiasm for the intervention or had other priorities for sustaining the intervention [56].

Furthermore, confusion in the roles and responsibilities about “who should do what” and “who should owe the program” were identified as barriers to sustainability in this study. Having clear accountability of roles and responsibilities were reported as key facilitators influencing the sustainability of hospital-based interventions according to a recent systematic review [57]. Sustainability depends not only on the capabilities of the schools to implement the program, nor the resources and material supports; but also on the targeted community’s involvement and a building sense of ownership [58].

### Implications of the study

Identifying the key predictive variables that helps to sustain the school-based SBCC intervention; the current study provided evidence that has practical implications for the school health promotion. The evidence could also help to EER schools and other stakeholders in malaria elimination efforts. More specifically, it has practical significance to ensuring the strategies of various national health policies that outlined the need to involve all those concerned and sustainability [3, 14, 15]. Therefore, exploring the stakeholders’ experiences and perceptions towards the adoption and sustainment of the school-based SBCC on malaria prevention, this study provided empirical evidence that supports various global malaria elimination initiatives.

Specifying a wide range of the SBCC components, assumptions, and activities that drew on extensive field experiences, the result of this study guides actions on how to embed such program within the existing health care, health education, and school activities to ultimately enhance ownership and sustainability. The measurements employed in this study to explore the perceived fidelity of implementation, degree of adoptions, and sustainability of the SBCC could provide methodological solutions to both the recently emerging implementation sciences and the SBCC theory [59]. Thus, this result contributes to fill gaps in evidence on the application of the SBCC approach to design, implement and sustain communication interventions on malaria preventive actions in resources limited settings. Finally, the lesson learned from the lived experiences and perspectives of the key stakeholders would help to devise effective methods to engage, empower and retain schools in malaria elimination efforts and beyond.

### Limitation of the study

The current study measured or predicted the anticipated sustainability of the school-based SBCC program based on the stakeholders’ perspective or based on subjectively defined data that were collected immediately after the cessation of the program. Despite its ultimate importance in implementation science, the actual sustainability of the program or the extent to which the program components are continued for some time after phase-out of the program was not explored in this study. Nevertheless, this study conceptualized sustainability as a process outcome of an intervention which acts as an intermediate indicator between the intervention and its desired effects and this is useful to understand the relationship between the intervention and ultimate effects [23].

Researchers argue that the need to consider sustainability during the design and implementation of interventions because program implementation and sustainability are parallel and concomitant processes [50]. Having an understanding of what processes (e.g training, planning, partnerships, engagement) and factors influence the sustainability of interventions would help to plan proactively for the continuation [60]. Applying the mixed research method, the current study explored various organizational systems and interpersonal factors influencing the anticipated sustainability of the program from the stakeholders’ perspectives. Ultimately the evidence from this study would help to confidently attribute the effects or changes in the malaria preventive psychographics and behavioral outcomes (i.e. ITN utilization and prompt care-seeking behaviors) to the intervention.

## Conclusion

The study identified key predictive variables such as stakeholders’ perceived risk to malaria, self-efficacy, perceived fidelity of implementation, and degree of adoption that could help to improve the sustainment of the school-based SBCC approach on malaria prevention and control. Further longitudinal study should be conducted to examine the rate of decline in program components over time and how improved sustainability would contribute to the effectiveness of malaria preventive behaviors among students.

## Data Availability

The datasets used and analyzed during the current study are available from the corresponding author on reasonable request.
